# Advances in Assessment and Training of Perceptual-Motor Performance

**DOI:** 10.3390/brainsci15070712

**Published:** 2025-07-02

**Authors:** Gary B. Wilkerson

**Affiliations:** Department of Health & Human Performance, University of Tennessee at Chattanooga, Chattanooga, TN 37403, USA; gary-wilkerson@utc.edu

Historically, efforts to optimize human health and performance have focused on the assessment and training of physical capabilities, such as muscle strength, flexibility, power, endurance, and speed. Recent technological advances in neuroimaging and electrophysiological testing have been revealing brain processing mechanisms that convert sensory inputs into coordinated interactions with the environment [1,2,3]. Consequently, interest has been growing in the potential for improvement in human performance and reduction in injury risk through training of modifiable cognitive processes that promote perceptual-motor coupling efficiency [4,5,6]. Improved understanding of neuromechanical mechanisms underlying goal-directed behaviors clearly has the potential to dramatically promote the effectiveness of innovative interventions for perceptual-motor performance deficiencies [7,8].

Daily life presents demands for deliberative, intuitive, and reactive responses to changing circumstances which vary in terms of utilization of prior experience stored in long-term memory and the urgency to take action. Competitive sports and certain work-related situations require extremely rapid perceptual-motor responses on a frequent basis, and everyone occasionally encounters scenarios that can have severe consequences unless an action is executed in a fast and accurate manner. Furthermore, emerging neuroscience evidence is identifying previously unknown interactions of brain processes with immune, autonomic, endocrine, digestive, and metabolic processes, which provide support for an exceedingly broad range of practical applications.

A strong foundational knowledge in neuroscience is not necessarily a prerequisite for understanding much of the content published in the *Brain Sciences* Special Issue on “Advances in Assessment and Training of Perceptual-Motor Performance.” The authors who contribute content are motivated to share evidence-based insights that will prove to be beneficial to readers who share the common goal to promote human health and well-being. In the realm of competitive sports, ranging from youth to professional levels, coaches, athletes, and sports medicine practitioners relentlessly seek improved methods that will maximize the potential for performance success. “Performance Neuroscience” [9] is a term that will undoubtedly be increasingly used to designate new approaches to the training and rehabilitation of athletes, as well as the general population. Another *Brain Sciences* Special Issue is currently under development to provide readers with additional information about the rapidly growing area of interest in brain–behavior relationships.
